# A combination therapy of Phages and Antibiotics: Two is better than one

**DOI:** 10.7150/ijbs.60551

**Published:** 2021-08-18

**Authors:** Xianghui Li, Yuhua He, Zhili Wang, Jiacun Wei, Tongxin Hu, Jiangzhe Si, Guangzhao Tao, Lei Zhang, Longxiang Xie, Abualgasim Elgaili Abdalla, Guoying Wang, Yanzhang Li, Tieshan Teng

**Affiliations:** 1 Institute of Biomedical Informatics, school of Basic Medical Sciences, Henan University, Kaifeng 475004, China.; 2 Henan International Joint Laboratory of Nuclear Protein Regulation, school of Basic Medical Sciences, Henan University, Kaifeng 475004, China.; 3 Department of Clinical Laboratory Sciences, College of Applied Medical Sciences, Jouf University, Sakaka 2014, Saudi Arabia.

**Keywords:** phage-antibiotic synergy, multidrug-resistance, bacterial anti-phage resistance, biofilm, phage therapy

## Abstract

Emergence of antibiotic resistance presents a major setback to global health, and shortage of antibiotic pipelines has created an urgent need for development of alternative therapeutic strategies. Bacteriophage (phage) therapy is considered as a potential approach for treatment of the increasing number of antibiotic-resistant pathogens. Phage-antibiotic synergy (PAS) refers to sublethal concentrations of certain antibiotics that enhance release of progeny phages from bacterial cells. A combination of phages and antibiotics is a promising strategy to reduce the dose of antibiotics and the development of antibiotic resistance during treatment. In this review, we highlight the state-of-the-art advancements of PAS studies, including the analysis of bacterial-killing enhancement, bacterial resistance reduction, and anti-biofilm effect, at both *in vitro* and *in vivo* levels. A comprehensive review of the genetic and molecular mechanisms of phage antibiotic synergy is provided, and synthetic biology approaches used to engineer phages, and design novel therapies and diagnostic tools are discussed. In addition, the role of engineered phages in reducing pathogenicity of bacteria is explored.

## Introduction

*Alexander Fleming* discovered the first antibiotic, penicillin in 1928 and this marked the beginning of the era of antibiotics 1. Various antibiotics are extensively used to fight infectious diseases in clinical practice. However, there has been a rapid increase in the levels of bacterial drug-resistance due to lack of effective control system and inappropriate use of antibiotics. World Health Organization (WHO) listed antibiotic resistance as one of the three most important public health threats in the 21^st^ century^2^. According to the estimates of economist *Jim O'Neill* who was commissioned by the UK Prime Minister, drug-resistant bacteria cause about 700,000 deaths worldwide every year 3. Furthermore, it is projected that 10 million and 300 million deaths directly and indirectly, associated with infections caused by drug-resistant bacteria will occur by 2050 respectively, exceeding the current number of cancer-related deaths 4. Therefore, it is likely that the post antibiotic era is slowly approaching and human beings are likely to face a world without effective antimicrobial drugs 5.

To address the looming threat of drug-resistant bacteria, scientists have proposed phage therapy as an alternative to antibiotic therapy. Phages also known as bacterial viruses, are widely distributed in nature and can infect and kill bacteria 6. Use of phages have been reconsidered as therapeutical tools due to the raised antimicrobial resistance (AMR) 7. Notably, the ability of phages to counteract multidrug-resistant bacteria has several advantages compared with antibiotics, including high specificity 8, 9, low dosage, low cost of production 10, 11, high safety and antibiofilm activity 12, 13. Instead of replacing antibiotics with phages, scientists have proposed that a combination of these two types of antibacterial agents may be more effective compared with use of either independently. In addition, the joint approach might confer possible advantages such as enhanced bacterial suppression, stronger effective penetration into biofilms and reduced capacity of bacteria to develop phage and/or antibiotic resistance. In this study, phage-antibiotic synergy refers to an increase in phage production following exposure to sublethal levels of bactericidal antibiotics 14, and this is considered a promising therapeutic strategy.

## Interactions between Phage and Antibiotic

Previous studies used various experimental models to determine the synergistic effect of various types of phages and antibiotics. These models include plaque assessments, elimination of drug- or phage-resistant bacteria, reducing the number of bacteria embedded in biofilm and *in vivo* evaluation^15^. This section focuses on the different evaluation methods. A summary of the types of phages and bacteria, as well as the synergistic effect of phage-antibiotic combinations and the corresponding references is presented in Table 1.

### PAS in Plaque assessments

The term “phage-antibiotic synergy” (PAS) was first coined by *Comeau AM et al* in 2007. In that first report, the researchers observed that stimulation by sublethal concentrations of *β*-Lactam and quinolone antibiotics resulted in significantly higher diameter and number of plaques, implying that a higher adsorption rate, shorter latent period and larger burst size occurred during plaque formation 14, 16. In addition, *Uchiyama et al* 17 screened 21 types of antibiotics with synergistic effect with *Pseudomonas aeruginosa* phage, most of which were effective in inhibiting bacterial cell wall synthesis or protein synthesis. A similar phenomenon of PAS was reported using species such as *Burkholderia cepacia* and *Staphylococcus aureus* by determining the plaque diameter 18, 19. Besides enlarging plaque size, antibiotics have been shown to significantly affect phage adsorption rates and the latent period during these infections. For example, *Ryan et al*. 20 treated* Escherichia coli* with either T4 phage alone or with a combination of cefotaxime and T4. The study findings showed that in absence of the antibiotic, the T4 phage had a latent period of 24 min which was reduced to 18 min upon addition of cefotaxime. Furthermore, the initial concentration of T4 phage increased from 5×10^6^ to 5×10^7^ plaque forming unit (PFU)/ml, indicative of a better replication as well as an increase in the rate of phage adsorption. Another study reported a significant increase in the burst size of *E. coli* phage ϕMFP following treatment with sublethal concentrations of cefotaxime, which was considered to be the cause for formation of long bacterial filaments 17. In summary, these findings show that addition of antibiotics causes changes in plaque diameter, latency period and burst size during growth of phages, and can therefore be used to determine the synergistic effect of phages and antibiotics.

### PAS in drug- or phage-resistant bacteria treatment

Drug-resistant bacteria pose a major threat to human health during clinical practice. Phage therapy is an alternative to antibiotics and studies report that it is effective in circumventing bacterial resistance. However, an important concern is that bacteria can also develop resistance to phages. Therefore, the recently proposed PAS is a potential approach for management of bacteria resistant to both antibiotics and phages. Several studies report that PAS can significantly reduce bacterial density, especially in phage and drug-resistant bacteria 21-24. In addition, PAS effectively limits production of bacterial virulence factors 25.

A previous study reported that 8 hours of treatment with a combination of 1/10 minimum inhibitory concentration (MIC) (0.05 mg/L) ciprofloxacin and phage ECA2 caused a significant decrease in the colony forming unit (CFU) of *E. coli* (decreased by about 7.8 folds). However, there was no significant decrease in bacterial counts following treatment with individual doses of the phage or antibiotic 26. Furthermore, phage-antibiotic combination treatments suppress pathogen activity and mitigate antibiotic or phage resistance in bacteria. T*orres-Barceló et al* 27 treated *P. aeruginosa* strain PA01 with either phage LUZ7 alone or a combination of streptomycin and LUZ7 and observed bacterial regrowth after 24 hours, which can be attributed to mutant phage-resistant subpopulations. However, a combination of streptomycin (100 and 240 mg/mL) with phage LUZ7 (10^5^ PFU/mL) effectively prevented development of phage-resistant PA01 mutants. Moreover, the combination therapy showed a 4-log CFU/ml loss of bacterial viability, compared with either antibiotic (100 and 240 mg/mL) or single-phage (10^5^ PFU/mL) therapy alone. Another PAS analysis was performed on a recombinant *P. aeruginosa* strain with plasmid pUCP24 that was resistant to gentamicin 28. The recombinant *P. aeruginosa* strain was administered with either a monotherapy and combination therapy consisting of gentamicin and filamentous phage Pf1. Analysis of vitality of overnight cultures containing approximately 10^6^ CFU, after exposure to 300 μg/ml gentamicin, showed that the growth of strain PA01 was not significantly affected. However, treatment with 50 μg/ml gentamicin and 10^8^ PFU/ml of Pf1 phage effectively inhibited growth 24.

### PAS in biofilm treatment

Bacterial biofilms refer to communities of microbes attached to an abiotic or biotic surface, such as medical implants, including catheters and artificial hip joints. The community of cells is encapsulated in an extracellular matrix comprising extracellular DNA, secreted proteins, lipids and polysaccharides that are collectively referred to as extracellular polymeric substances (EPS). These substances allow adhesion to the surface and provide protection against antimicrobial agents 29, 30. However, biofilms represent a dangerous reservoir for persistent bacteria, which is a phenotypic variation in bacterial population and confers significant antimicrobial resistance without genetic mutations and drug resistance genes 31-33. Therefore, biofilms are more difficult to eradicate compared with planktonic bacteria treated with only antibiotic therapy. As a result, biofilms are considered as a potential non-antibiotic therapy. Notably, numerous studies have explored the combined effect of phages and antibiotics for elimination of bacteria in biofilms 16, 20.

For instance, a previous study explored efficacy of phage Sb-1 alone or in combination with different classes of antibiotics for elimination of *S. aureus* biofilms in a rat model 34. The findings showed that treatment with phage Sb-1 alone significantly reduced persistent bacteria although it did not eradicate the biofilm. However, simultaneous treatment with phage Sb-1 and rifampicin/daptomycin significantly degraded EPS and eradicated *S. aureus* biofilm. Moreover, staggered phage Sb-1 and antibiotic treatment is an effective strategy for degradation of biofilms.

After bacterial infection, toxic genes encoded by phage genomes are expressed as specific enzymes, such as peptidoglycan hydrolase and polysaccharide depolymerases, which are involved in cleavage of bacterial peptidoglycans. A combination of hydrolytic enzymes, encoded by phages, with specific antibiotics significantly destroys the structure of bacterial biofilms and releases persistent bacteria embedded in the biofilm into the nutrient environment. This subsequently enhances metabolic activity of persistent bacteria in the nutrient environment, making them more sensitive to antibiotics 5, 35-37. For instance, a 6-hour treatment with a combination of KPO1K2 and ciprofloxacin significantly eliminated a biofilm formed by *Klebsiella pneumoniae*, and caused a 4.5-fold reduction in the number of bacteria embedded in the biofilm.

### PAS in animal models

Evaluating effects of PAS *in vitro* has a number of limitations due to lack of an immune effect 38. Several studies have explored the synergistic effect of phage-antibiotic combinations *in vitro*, but not *in vivo* 39-41. A previous study simulated bacteremia and bladder infection conditions, and reported that serum and urine components completely prevented PAS between phage φHP3 and ceftazidime, by determining the bacterial density which was significantly decreased in the culture medium. This finding indicates that human host conditions suppress PAS on bacterial growth *in vivo* 42.

Furthermore, animal models can be used to evaluate the effect of phages in weakening inflammatory reaction, in order to ascertain the exact synergistic effect of phages and antibiotics. Results from previous animal models show that phage infection triggers a specific immune response, although other studies report that phages cannot cause significant disease symptoms *in vivo*^43^, ^44^. Studies report that liposome-encapsulated phages can significantly alleviate inflammatory responses, thereby providing a basis for phage treatment *in vivo*. For example, *Kaur et al*. 45 reported that levels of PCT, IL-1β and TNF-α cytokines in mice by K-wire, surgically implanted into the intra-medullary canal, implanted with a specific phage and linezolid were lower compared with those in a K-wire embedded with phages or antibiotics only. Consequently, the number of intracellular bacteria will be reduced to a certain level, increasing effectiveness of oxidative killing by phagocytes 46. In this case, the phagocytic function and bactericidal activity of macrophages is significantly enhanced since bacteria are phagocytized by macrophages *in vivo*. Notably, the pathogen can also rapidly develop resistance to phages *in vivo*. However, previous studies report that neutrophils are more effective in scavenging phage-resistant, compared with phage-sensitive, bacteria 38. Moreover, *Tiwari et al*. 47 reported that phage PA1Ø significantly reduces the lethal rate infected with immune-competent mice compared with neutropenic mice. Notably, use of phages for treatment of bacterial infections *in vivo* may produce high amounts of residue along with bacterial dissolution, including, lipopolysaccharides, cytoplasmic proteins, membrane particles and large pieces of cell debris 48. Therefore, it is not clear which components directly elicit an immune response in the body. Although some studies report that the level of inflammatory factors decreases after phage therapy, it is possible that this phenomenon may also be caused by reduction in the number of pathogens *in vivo* 49.

### PAS in clinical cases

Although several studies have discussed the effect of combining phages with antibiotics *in vitro* and *in vivo*, there were also some successful clinical experiments.* Bao et al* had reported a case of patient who developed a recurrent urinary tract infection (UTI) with extensively drug-resistant *Klebsiella pneumoniae* (ERKp) which resisted all tested antibiotics, except tigecycline and polymyxin B 67. After critical care treatments, including tigecycline administration, the UTI was not cured and became persistent. The patient was enrolled in phage therapy clinical trial after involvement evaluation and informed consent. *In vitro*, the combination of phage cocktail III (KP152, KP154, KP155, KP164, KP6377 and HD 001) and SMZ-TMP could completely suppress the growth of ERKp for more than 24h. Therefore, after treated with the above therapeutic regimen, including oral administration of trimethoprim-sulfamethoxazole (SMZ-TMP) twice a day, and bladder irrigation with phage cocktails III for five days of continuous treatment, the ERKp couldn't be isolated from the patient's urine, and the symptoms of urinary tract infection disappeared completely. Moreover, there was no sign of recurrence within six months after discharge.

A case of renal transplant patient developed urinary tract infection with an extended-spectrum *β*-lactamase (ESBL)-positive *K. pneumoniae* strain in the first month post-transplant was ineffective in the treatment of multiple antibiotics 68. Although ESBL *K. pneumoniae* in this case was sensitive to meropenem, the infection recurred eventually evolved into epididymitis after repeated treatment with meropenem. A phage from Georgia exhibited excellent lytic activity against this ESBL-*K. pneumoniae* isolates. After treatment with meropenem combined with this phage by oral and bladder irrigation, respectively, the urethral symptoms of the patient completely subsided within one day, and urine cultures remained negative for 14 months after treatment.

The phage OMKO1's receptor is the outer membrane protein M of mexAB- and mexXY-multidrug efflux systems of *P. aeruginosa*, which is essential for antibiotic (eg. ceftazidime and ciprofloxacin) pump-out, thus OMKO1 induced receptor-mutant-resistant strain would be more susceptible to ceftazidime. In a case of therapeutic application of phage OMKO1 to treat a drug-resistant *P. aeruginosa* infection of an aortic Dacron graft, *Benjamin K. Chan et al*. conducted an experiment *in vitro* and found that both phage OMKO1 alone and ceftazidime combined with phage OMKO1 could reduce bacterial density. Especially ceftazidime at 2×MIC + phage OMKO1 treatment could significantly reduce the bacterial density, compared with ceftazidime alone in the treatment of bacterial biofilm infection 66. In addition, the OMKO1 (10^7^ PFU/ml) and ceftazidime (0.2 g/ml) solution was successfully used in the treatment of patients with aortic perforation infection, and the patient represented stable vital signs and there was no sign of recurrent infection for 18 months.

### Phage-antibiotic antagonism

Efficient synergistic bactericidal effects are achieved when these phages are combined with specific antibiotics. Additionally, other phage-antibiotic combinations show no synergistic or antagonistic effects 69 (Table 2). A typical example is rifampicin, which can inhibit the growth of host bacteria by targeting its RNA polymerase. Rifampicin also inhibits the production of phage particle, due to the fact that its replication depends on the host RNA polymerase. This function of rifampicin is shown the antagonistic effect with a number of phages (Table 2). However, when the phage encodes its own RNA polymerase for virion replication rather than the host bacteria's, Rifampicin will not restrain the production of progeny phage, which shows the synergistic bactericidal activity of phages-antibiotics. For instance, *P. aeruginosa* phage ϕKZ could encode its own RNA polymerase, infect the host bacteria and produce the progeny phage in the presence of 400 µg/mL of rifampin 70. However, one counter-example, the virion replication of phage LUZ19 depending on the host RNA polymerase was completely inhibited at the same concentration of rifampicin. These results indicate that the interaction effects between phages and antibiotics depended on the type of antibiotics or phages 71.

## Genetic mechanism underlying PAS

Initial infection with phages occurs through binding to receptors, such as lipopolysaccharides, teichoic acids, proteins, and flagella, on the surface of bacteria. Although emergence of phage-resistant bacteria is likely inevitable, numerous studies report that phage selective pressure may accelerate bacterial mutations thus promoting them to subvert phage infection, but with a cost to their fitness 90. Such fitness trade-offs include reduced virulence, limited nutrient uptake, resensitization to antibiotics, and colonization defects. This observation lays a basis for application of phages. In addition to direct killing effect of phages on host bacteria, selective pressure produced by phages is useful in limiting bacterial growth. Surface molecules of bacteria play an important role in disease phenotypes, in a similar mechanism to receptor molecules on phages 91, 92. These surface components consist of lipopolysaccharides (LPS), outer membrane proteins, teichoic acid, type IV pili, capsules, siderophores receptors and the efflux pumps. Their components are often considered to be virulence factors, antibiotic resistance related factors and normal growth factors, as they can mediate attachment to and damage of hosts and antibiotic efflux, respectively 93-95.

The ferric catecholate receptor, known as FepA on the surface of *Salmonella enterica* is the key protein for siderophore mediated iron transport in bacteria. However, FepA can also act as a bacterial receptor, playing a role in adsorption of phage H8. Notably, the gene encoding FepA mutates to resist phage infection under high pressure of phage selection. On the other hand, its mutant strain cannot transport iron from the environment, causing its own death due to growth restriction 96. Previous studies report that phage H8 can force a desired genetic trade-off between phage resistance and growth restriction, a phenomenon that can be beneficial in phage therapy against MDR-*S. enterica*.

The outer membrane porin M (OprM) in *P. aeruginosa* is an indispensable component of drug efflux systems including MexAB-OprM and MexXY-OprM. For example, *Chan et al.* 97 reported that OprM can be recognized by phage OMKO1 as a key receptor protein. In addition, the gene encoding OprM mutates under selection pressure during phage infection, thus allowing *P. aeruginosa* to tolerate phage OMKO1 during chronic infection. Moreover, OprM mutation simultaneously causes a deficiency in drug efflux pumps, thus preventing elimination of antibiotics. In summary, these results indicate that phage infection triggers an evolutionary trade-off in *P. aeruginosa*, where the evolution of bacterial resistance to phages interferes with the function of efflux pumps, thus increasing sensitivity to antibiotics.

**Figure 1:**
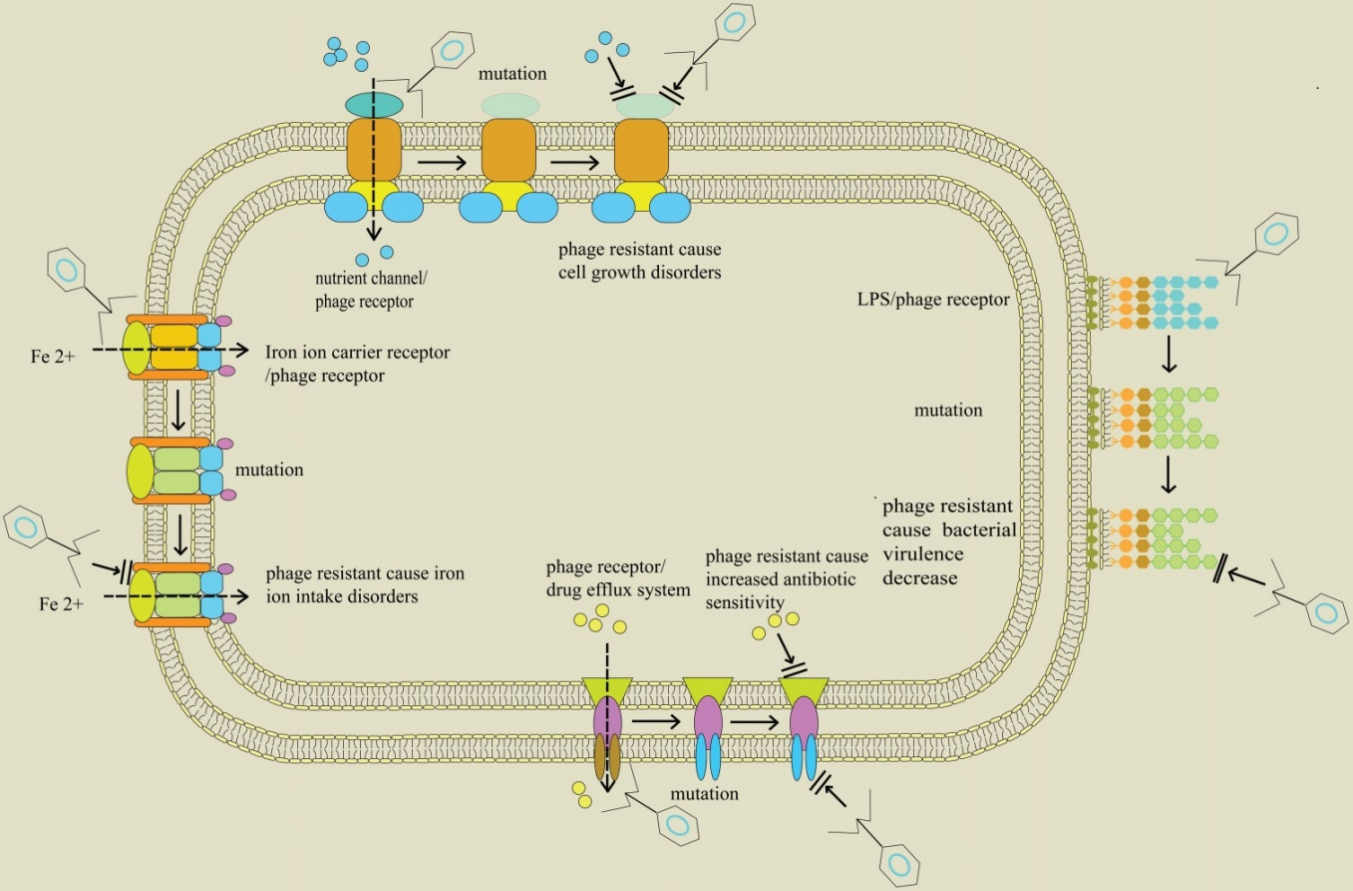
Trade-off in bacteria between phage resistance and bacterial fitness; Profile of proteins found on the surface of bacteria including nutrient channels, lipopolysaccharide (LPS), drug efflux pumps, and siderophore receptors. They are related to bacterial life history traits and initial infection of phage. When the genes encoding these proteins are altered, through events such as mutations, the bacteria exhibit the characteristics of phage resistance. In addition, these changes separately block bacterial intake of nutrients, downregulate virulence factors, and hinder entry of iron ion into bacteria, thus affecting normal growth of bacteria. Moreover, the blocked drug efflux system predisposes bacteria to antibiotics.

Furthermore, *ompU* gene which encodes the outer membrane porin in *Vibrio cholerae* and acts as a phage infection receptor in the bacteria, produces evolutionary selection pressure as a result of phage infection 98. Under this selective pressure, *ompU* or *toxR* (regulatory gene of *ompU* expression) genes in *V. cholerae* mutate, resulting in resistance to phages. Similarly, *V. cholerae* displays an evolutionary trade-off between phage resistance and bacterial virulence. Notably, these mutations attenuate virulence by at least 100-fold since the mutant strains are unable to cause cholera and loses the ability of disease transmission. These results indicate that adaptation to phage infection involves trade-offs in evolutionary fitness and provides a molecular basis for understanding the effect of phage infection on transmission of *V. cholerae* as well as seeding of environmental reservoirs (Figure 1). The findings from these studies show that phages in combination with antibiotics synergistically act against host bacteria and alter expression of bacterial virulence factors, antibiotic resistance and activity of growth factors. These mechanisms, in turn, cause an increase in antibiotic sensitivity or inhibition of bacterial growth (Table 3).

## Use of engineered phages in enhancing susceptibility to antibiotics

The current rapid advances in sequencing technology and molecular biology have led to increase in development of genetically-engineered phages. Genetically-engineered phages are effective in eliminating drug-resistant pathogens and provide a key therapy for treatment of patients 5, 106, 107. The recombinant phage can efficiently restore sensitivity of drug-resistant bacteria, decrease MIC value of antibiotics and target deletion of essential genes in host bacteria. Notably, several studies have reported successful construction of genetically-engineered phages that exhibit significant activity against drug-resistant bacteria (Table 4).

Previous studies report that the genome of phage M13 can be edited to overexpress the SOS inhibitor, LexA3 108. Consequently, the modified M13 inhibited SOS reaction following DNA damage to the bacteria. In addition, the bactericidal effect of the modified M13 combined with antibiotics was significantly augmented. A combination of modified M13 and Ofloxacin showed a 2.7-fold increase in the bactericidal effect and significantly reduced bacterial resistance, compared with single-dose antibiotic treatment 109. A previous study inserted a streptomycin sensitive gene into the genome of phage lambda (*λ*). Treatment of a streptomycin-resistant *E. coli* with this recombinant phage restored their sensitivity to the antibiotic, and the MIC value of the antibiotic against *E. coli* decreased from 100 to 12.5 mg/ml. The same method was used to restore sensitivity of *E. coli* to nalidixic acid, achieving a 2-fold reduction in MIC value 110.

Clustered Regularly Interspaced Short Palindromic Repeat (CRISPR)/Cas9 system is an adaptive immune mechanism formed by bacteria during their evolution. Currently, CRISPR/Cas9 nuclease has gained popularity as a major tool for targeted deletion of foreign DNA in pathogens. In a previous study, researchers integrated the genome of phage *λ* with the CRISPR/Cas9 system to target genes encoding *β*-lactamases in the *E. coli* genome, then infected *E. coli* resistant to *β* lactams with the modified phage *λ*. The results showed that the drug-resistant *E. coli* regained its sensitivity to the antibiotics 111. In a similar study,* Park et al*. 112 used Cas9-triggered homologous recombination to integrate a CRISPR/cas9 targeted NUC gene (nuclear gene common to all *S. aureus*) into the genome of phage ØSaBov. Infecting* S. aureus* with the recombinant phage resulted in death of all bacteria within eight hours *in vitro,* whereas the number of pathogens *in vivo* reduced by 2-fold 112.

## Discussion

A combination of phages and antibiotics has been extensively used to enhance eradication of drug-resistant pathogens, and alleviate the widespread antibiotics resistance worldwide 133. Numerous experimental models, including plaque analysis, liquid plankton, biofilm tests and animal experiments, have been used to successfully evaluate the synergistic effect of phages and antibiotics. Several studies have explored the underlying mechanism of synergy between phages and antibiotics. Notably, the seesaw effect of host evolution explains the mechanism of this synergistic bactericidal effect 15. Phage infection can exert selective pressure on bacteria, thus predisposing them to gene mutations 134. Under this selective pressure, there is loss or down-regulation of some of the host bacteria's important components related to bacterial toxicity, drug sensitivity and growth factors. Studies report that phage-resistant strains exhibit lower toxicity, are more sensitive to antibiotics and have slower growth rate compared with wild strains 12.

## Figures and Tables

**Table 1:** Phage-antibiotic combinations with confirmed positive interactions

Pathogens	Strains	Antibiotics	Phage	Synergistic effects	Ref.
**Plaque assessments**					
*S. aureus*	-	AMX	ϕSZIP1	++	18
	-	TIM	ϕSZIP1	+	
	-	CRO, CHL	ϕSZIP1	+	
*P. aeruginosa*	PA5	CP, CPZ, FOM, etc.	KPP21	+++	17
	PAO1, PA4, PA23, etc.	AMK, AZT, CAZ, etc.	KPP22	+++	
	PA3	CPZ/SBT, FOM, TOB	KPP23	+++	
*B. cepacia*	K56-2/C6433	CIP, MEM, TET	KS12/KS14	+++/++	19
*E. coli*	MFP/	AZT, CFM, CTX, etc.	ϕMFP/RB32/ RB33/T4	+	50
*E. coli*	ATCC11303	AML, AMP, CFR, etc.	Φszut/ ϕSZIP1/ϕSZIP2	+	20
**Drug- or phase-resistant bacteria/biofilm**					
*E. coli*	ATCC 11303	TOB	T4	+^b^	22
	ATCC 13706	CIP	ECA2	+++^a^	26
	JE2571	KAN, RIF	RPD1(T)	+^a^	51
*S. aureus*	PS80	GEN	SA5	+^a^, +^b^	52
	MRSA	TEC	Sb-1	+^a^, +++^b^	53
	MRSA	RFP/AZI	SAP-26	+^a^, +^b^	23
	MRSA	CIP, TET	PYO	^+a^	24
*P. aeruginosa*	PAO1	CAR, GEN, TET, etc.	Pf3	+	24
	PAK	CAR, GEN, CHL	Pf1	+	
	ATCC 9027	CRO	σ-1	++^a^	54
	PA-4U	CRO	δ	+^a^	
	PA-M2	CRO	001A	++^a^	
	PAO1	STR	LUZ7	+^a^	27
	PA01/PAPS	TET, ERY, CIP, etc.	OMKO1	++^a^	21
	CHA	CIP, MEN	Cocktail	++^a^	55
	PAO1	CAZ, CIP	LKD16	++^a^	56
	PAO1	CAZ, CIP	LUZ7,14/1	+^a^	
	PAO1	CAZ, PIPC	KPP22	++^a^	57
	PA14	CAZ, CIP, GEN, etc.	NP1, NP3	+^a^	35
	PAO1	CST	KTN4 (M)	+^a^	56
	PA365707, PA364077	CIP	PEV20	++^b^	58
*A. baumannii*	AB01, AB04, AB16	MEM, CIP, MEM	KARL-1	++^a^	59
*K. pneumoniae*	KPB5055	CIP	KPO1K2	+^b^	60
	KPB5055	AMX	Not known	++^a/b^	61
*B. cepacia*	K56-2	CIP, TET, MEM	KS12	+^a^	19
***In vivo***					
*E. coli*	poultry isolate	ENR	SPR02/DAF6	+++	62
*S. aureus*	ATCC43300	LZD	MR-10	+	63
*P. aerginosa*	CHA	CIP	cocktail	++	55
*B. cepacia*	K56-2	MIN	KS12	+	19
	K56-2	MEM	KS12	++	
*E. faecalis*	V583	AMP	EFDG1, EFLK1	+++	64
*K. pneumoniae*	KPB5055	AMK	SS (P)	+	65
**Clinical cases**					
*P. aerginosa*	-	CAZ, CIP	OMKO1	*	66
*K. pneumoniae*	ERKp	SMZ-TMP	KP152, KP154, KP155, KP164, KP6377, HD001	*	67
*K. pneumoniae*	ATCC 700603	MEM	unknown	*	68

“a” represents the effect on planktonic bacteria, “b” represents the effect on biofilms. “-” represents an unknown species of bacteria used, “*” represents clinical trials, “+” shows that the percentage of phage-antibiotic synergistic effect is enhanced compared to phage alone and its number represents the degree of enhancement. “+” indicates 10~50%, “++” indicates 50~80% and “+++” indicates above 80%. The names of antibiotics are abbreviated as follows: Amikacin [AMK], Ampicillin [AMP], Amoxicillin [AMX], Azithromycin [AZI], Aztreonam [AZT], Carbenicillin [CAR], Ceftazidime [CAZ], Cefixime [CFM], Chloramphenicol [CHL], Ciprofloxacin [CIP], Cefoperazone [CPZ], Sulbactam/Cefoperazone [SBT/CPZ], Ceftriaxone [CRO], Colistin [CST], Cefotaxime [CTX], Enrofloxacin [ENR], Erythromycin [ERY], Fosfomycin [FOM], Gentamicin [GEN], Kanamycin [KAN], Linezolid [LZD], Meropenem [MEM], Minocycline [MIN], Piperacillin [PIPC], Rifampicin [RIF], Streptomycin [STR], Teicoplanin [TEC], Tetracycline [TET], Ticarcillin [TIM] and Tobramycin [TOB].

**Table 2:** No synergistic or antagonistic effects of the combination of phage and antibiotic

Pathogens	Antibiotics	Phages	Antagonistic effects	Refs.
*M. tuberculosis*	RIF, INH	TM4, D29	Inhibiting production of phage particle	72-76
*P. aeruginosa*	RIF	LUZ19	Inhibiting production of phage particle	70
	CIP, MEM	Phage cocktail	No synergistic	55
	CIP, TOB, GEN	NP1, NP3	Inhibiting production of phage particle	35
*B. subtilis*	RIF	SPO1	Reducing bacteriolytic activity	77
	RIF	β22, AR9	Inhibiting production of phage particle	78, 79
	Nalidixic Acid	SP50, SP82, etc.	Inhibiting production of phage particle	80
*E. coli*	RIF	λvir, T2, T5, Mu	Inhibiting production of phage particle	81-84
	Nalidixic Acid	ϕR, T2, T7, etc.	Inhibiting production of phage particle	85, 86
	CHL, TET	ECA2	Reducing bactericidal activity	26
	CIP	ELY-1	Inhibiting production of phage particle	87
*P. aeruginosa*	RIF	PM2	Inhibiting production of phage particle	88
*R. solanacearum*	RIF	ΦRP12, ΦRP31, ΦRSB1, etc.	Inhibiting production of phage particle	89
*S. aureus*	GEN, RIF, LZD, etc.	PYO	Inhibiting production of phage particle	24

**Table 3:** Phage-induced changes in bacterial fitness and antibiotic resistance

Pathogens	Phage	Target/Effect	Result	Ref.
*Salmonella*	f2αSE, f3αSE, f18αSE	LPS/phage receptor, virulence factor	Attenuating virulence	99
*Salmonella*	φ1	LPS/phage receptor, virulence factor	Attenuating virulence	100
*V. cholerae*	ICP2_2013 _A_Haiti	OmpU/phage receptor, virulence factor	Attenuating virulence	98
*E. coli K-1*	NM	OmpA/phage receptor, immune system evasion	Immune system evasion	101
*S. aureus*	NM	Teichoic acids/phage receptor, virulence factor	Attenuating virulence	102
*S. aureus*	M^Sa^	Teichoic acids/phage receptor, virulence factor	Attenuating virulence	103
*B. cenocepacia*	NM	FepA/phage receptor, siderophore receptor	Inhibiting bacterial growth	104
*Salmonella*	H8	Siderophore transporter flagellum/phage	Inhibiting bacterial proliferation	96
*V. anguillarum*	Lambda Zap II	receptor, motility, virulence factor	Reducing motility	105
*P. aeruginosa*	OMKO1	OprM/phage receptor, efflux pump	Increasing sensitivity to antibiotics	97
*S. aureus*	SA5	Not known	Reducing antibiotic resistance	52
*P. aeruginosa*	LUZ7	Not known	Increasing sensitivity to antibiotics	27

**Table 4:** Bactericidal effect of genetically engineered phage

Stain	Phage	Method	Result	Refs.
*L. monocytogenes*	PSA	Removing lysogen module	Improving lytic ability	113
*L. monocytogenes*	B025	Removing lysogen module	Improving lytic ability	113
*S. aureus*	ØSaBov	Integrating with CRISPR/Cas	Improving lytic ability	112
*S. aureus*	Φ11	Recombining wit SnCe6	Improving lytic ability	114
*C. albicans*	JM	Recombining with PPA	Improving lytic ability	115
*E. coli*	M13	Recombining with CAP	Improving lytic ability	116
*E. coli*	M13	Overexpressing LexA3	The synergetic bactericidal efficacy of engineered phage M13 and ofloxacin was increased by 2.7 logs	109
*E. coli*	*λ*	Recombining with streptomycin sensitive genes	The synergetic efficacy of engineered *λ* and streptomycin reduced MIC value from 100 g/ml to 12.5 g/ml	110
*E. coli*	*λ*	Integrating with CRISPR/Cas	The synergetic efficacy of *λ*_Cas-CRISPR_ and streptomycin sensitized and killed antibiotic-resistant bacteria	111
*E. coli*	T7	Recombining with Dsp8	Improving lytic ability	117
*E. coli*	M13	Recombining with toxin gene	Improving lytic ability	118
*P. aeruginosa*	T7	Recombining with AILA	Improving lytic ability	119
*C. trachomatis*	M13	Recombining with RGD and PmpD	Improving lytic ability	120
*E. coli*	T2	Recombining with tail fiber	Expanding phage host range	121
*E. coli*	FD	Rcombining with IKE	Expanding phage host range	122
*E. coli*	T3	Replacing tail fiber gene 17	Expanding phagehost range	123
*H. pylori*	M13	Rcombining with gene 3 protein	Expanding phage host range	124
*E. faecalis*	ΦEf11	Reorganizing with defective ΦFL1C	Expanding phage host range	125
*E. coli*	T2	Replacing host recognition genes	Expanding phage host range	126
*L. monocytogenes*	A511	Bacteriophages PEGylation	Enhancing the half-life of phage	127
*S. typhi*	Felix-O1	Bacteriophages PEGylation	Enhancing the half-life of phage	127
*E. coli*	T7	Inserting PhoE signal peptide	Enhancing the half-life of phage	128
*S. aureus*	P954	Inserting cat phage genome	Reducing endotoxin production	129
*E. coli*	M13	Recombining with BgIII	Reducing endotoxin production	130
*E. coli*	M13	Recombining with Gef and ChpBK	Reducing endotoxin production	131
*P. aeruginosa*	Pf3	Recombining with endonuclease	Reducing endotoxin production	132
